# Genetic Association of Interleukin 33/ST2 Polymorphisms With Behcet’s Uveitis

**DOI:** 10.3389/fimmu.2021.589639

**Published:** 2021-03-25

**Authors:** Minghang Pei, Xinshu Liu, Peizeng Yang, Chan Zhao, Fei Gao, Yi Qu, Anyi Liang, Junyan Xiao, Meifen Zhang

**Affiliations:** ^1^ Department of Ophthalmology, The First Affiliated Hospital of Zhengzhou University, Zhengzhou, China; ^2^ Department of Ophthalmology, The Fourth People’s Hospital of Shenyang, Shenyang, China; ^3^ The First Affiliated Hospital of Chongqing Medical University, Chongqing Key Lab of Ophthalmology, Chongqing Eye Institute, Chongqing Branch of National Clinical Research Center for Ocular Diseases, Chongqing, China; ^4^ Department of Ophthalmology, Peking Union Medical College Hospital, Chinese Academy of Medical Sciences and Peking Union Medical College, Beijing, China

**Keywords:** Behcet’s disease, Behcet’s uveitis, uveitis, gene polymorphism, single nucleotide polymorphism, interleukin 33, ST2

## Abstract

Interleukin (IL)33, a member of the IL1 superfamily, functions as a nuclear factor and mediates biological effects by interacting with the ST2 receptor. Recent studies have described IL33 as an emerging pro-inflammatory cytokine in the immune system, and *IL33*/*ST2* gene polymorphisms have been implicated in the pathogenesis of various immune diseases. However, the underlying mechanisms of *IL33/ST2* in Behcet’s disease (BD) remain to be defined. Here, we investigated the association between *IL33/ST2* gene polymorphisms and BD in 585 BD uveitis (BDU) patients and 834 healthy controls using Agena MassARRAY iPLEX platform. We found that rs3821204 was associated with the development of BDU. Moreover, the frequency of rs2210463 G allele was lower in patients with genital involvement. Association analysis revealed a much greater genetic difference between complete-type and incomplete-type BD groups, including three SNPs (rs7044343, rs1048274, and rs2210463). Our findings suggest that *IL33/ST2* gene polymorphisms are involved in the pathogenesis of BDU. Different genetic backgrounds may exist in complete-type and incomplete-type BD patients.

## Introduction

Behçet’s disease (BD) is a chronic, relapsing, systemic vasculitis involving complex pathologic processes characterized by recurrent episodes of uveitis, oral and genital ulcers, and skin lesions (1). It may also affect the digestive, cardiovascular, and nervous systems and involve joints and large vessels (1). Eye involvement is one of the most serious manifestations of BD with high incidence of disability. It affects about 70% of patients and is characterized by uveitis (BDU) (2). The onset of BD usually occurs in the third or fourth decade of life and is rare in children or individuals over the age of 40 years. The etiology of BD is still unclear, although, an inflammatory response triggered by immune and unknown environmental factors in a genetically susceptible individual has been proposed as its cause. Among all genetic factors, HLA-B51 has been confirmed as the strongest risk factor for BD. However, the presence of HLA-B51 alone is not sufficient to explain all the symptoms of BD patients (3).

Interleukin (IL)33, a member of the IL-1 family, is evolutionarily conserved in mammals and a ligand for the orphan receptor ST2. It is constitutively expressed by tissue barrier cells, such as epithelial and endothelial cells, and various innate immune cells, such as macrophages and dendritic cells. Both enhanced innate immunity and neutrophil hyperactivity with endothelial damage, which are part of BD pathogenesis (4), are related to disrupted IL33 levels. Therefore, we hypothesized that BD may be associated with *IL33/ST2* genetic abnormalities.

In the present study, we aimed to establish the role of *IL33* and *ST2* genes in the risk of developing BDU in a well-characterized Chinese cohort by genotyping and association analysis. We analyzed single nucleotide polymorphisms (SNPs) of *IL33* (rs1891385, rs2210463, rs11792633, rs7044343, rs1048274) and *ST2* (rs3821204, rs12712142, rs2058660) with respect to BDU pathogenesis and characteristic BDU phenotypes in these patients.

## Materials and Methods

### Patients

This study was approved by the Ethics Committee of Peking Union Medical College Hospital. A total of 585 patients with BDU were recruited from the Peking Union Medical College Hospital (PUMCH, 190 patients) and the First Affiliated Hospital of Chongqing Medical University (395 patients) between January 2018 and June 2019. All patients with BD were diagnosed according to the International BD Study Group criteria. Eight hundred and thirty-four healthy controls with no previous history of immune-related diseases were enrolled from the health examination center of PUMCH. All participants were self-reported as Han Chinese and were unrelated.

Of the 585 BD patients included, detailed clinical data for 553 patients was recorded. Based on their clinical characteristics, patients were divided into the following subgroups: complete-type and incomplete-type BD groups (1); severe and non-severe BD groups; early-onset and late-onset BD groups.

In case of complete-type BD, all four main symptoms (ocular lesion, recurrent aphthous ulcers on the oral mucosa, genital ulcers, and skin lesion) appeared during the clinical course. In case of incomplete-type BD, either of the following conditions was met: 1. Three of the four main symptoms, or two main symptoms and two additional symptoms (arthritis without deformity or sclerosis, epididymitis, gastrointestinal lesion represented by ileocecal ulceration, vascular lesions, and central nervous system lesions including and above middle class of severity) appeared during the clinical course; 2. Typical ocular lesions and other main symptoms, or two additional symptoms appeared during the clinical course.

Severe and non-severe BD were classified based on clinical severity score ≥7 or <7 points, respectively. Clinical severity score was determined as previously described (5, 6): 1 point for each of the mild symptoms (oral ulcers, genital ulcers, arthralgia, typical skin lesions such as erythema, nodosum-like lesions, papulopustular lesions, and folliculitis), 2 points for each of the moderate symptoms (arthritis, deep vein thrombosis of the legs, anterior uveitis, gastrointestinal involvement), and 3 points for each of the severe symptoms (posterior/panuveitis, retinal vasculitis, arterial thrombosis, neuro Behcet’s disease, bowel perforation).

In case of early-onset BD, the age of first BD attack was <40 years, while in late-onset BD group, the age of first BD attack was ≥40 years.

### Selection of SNPs

Considering the vital effects of IL33 and ST2 in immune-mediated diseases, eight SNPs (rs1891385, rs2210463, rs11792633, rs7044343, rs1048274, rs2058660, rs3821204, rs12712142), which were reported to show a positive association with systemic lupus erythematosus, ankylosing spondylitis (AS), or other immune-related diseases, based on GWAS and candidate gene association studies, were selected for further analysis.

### Sample Collection and SNP Analysis

A total of 2 ml blood sample was collected in an ethylenediaminetetraacetic acid (EDTA) anticoagulant tube from each subject. Genomic DNA was extracted from peripheral blood cells using a kit from Thermo Fisher Scientific Inc. according to the manufacturer’s instructions. DNA concentration was determined by using a Nanodrop spectrophotometer (Thermo, Wilmington, DE, USA) using the 260/280 nm ratio and diluted to a working concentration of 15–20 ng/μl. The SNPs of *IL33* and *ST2* were genotyped using Agena MassArray system (Agena iPLEX assay, San Diego, CA, USA) following the manufacturer’s instructions. Both polymerase chain reaction (PCR) and single-base extension primers for *IL33* (rs1891385, rs2210463, rs11792633, rs7044343, rs1048274) and *ST2* (rs3821204, rs12712142, rs2058660) were designed by the Assay Design Suite (ADS, Agena). After multiplex PCR amplifications, the products were used for locus specific single base extension reactions and the final products were desalted and transferred on to a 384-element SpectroCHIP array. Allele detection was conducted by matrix-assisted laser desorption ionization time of flight mass spectrometry (MALDI-TOF MS). The mass spectrogram data were analyzed using MassArray Typer 4.0 software (Agena).

The current SNP assay method is based on primer extension and offers two levels of specificity. First, a locus-specific PCR reaction takes place, followed by a locus-specific primer extension reaction (iPLEX assay) in which an oligonucleotide primer anneals immediately upstream of the polymorphic site being genotyped. In the iPLEX assay, the primer and amplified target DNA are incubated with mass-modified dideoxynucleotide terminators. The primer extension is made according to the sequence of the variant site, and is a single complementary mass-modified base. Through the use of MALDI-TOF mass spectrometry, the mass of the extended primer is determined. The primer’s mass indicates the sequence and, therefore, the alleles present at the polymorphic site of interest (7).

Duplicate samples, negative controls, and blank controls were included on the plates to ensure the accuracy of genotyping.

### Statistical Analysis

Statistical analyses were performed using PLINK 1.07 software (Shaun Purcell, Boston, MA, USA). The Hardy-Weinberg equilibrium (HWE) of each SNP was examined by using the Chi-square (χ^2^) test in healthy controls. Any SNPs that deviated from the HWE (P < 0.05 in the control groups) would be excluded from subsequent analysis. Differences in genotype and allele frequencies between cases and controls, and between different BD subgroups and phenotypes were analyzed using χ^2^ test. The odds ratio (OR) and 95% confidence interval (95% CI) of associations were calculated. For multiple comparisons, Pc values (corrected for multiple comparisons by the Bonferroni adjustment test) <0.05 were deemed to be statistically significant. For genetic model testing (additive, dominant, and recessive model), logistic regression analyses were used.

## Results

### Patient Characteristics and Sequencing Analysis

The characteristics of the controls and patients with BD are shown in **Table 1**. The gender distributions in both controls and cases were similar (p = 0.127). The median age was 36.0 (31.0–42.0) years and 32.0 (26.0–39.0) years for controls and cases respectively. The success rate for sequencing of each SNP was more than 99%. The eight SNPs of *IL33* and *ST2* selected in the present study did not show any deviation from HWE (all P > 0.05) in controls, and thus, the data were suitable for further comparisons.

**Table 1:** Clinical and demographic characteristics of the study population.

Characteristic	Patients with BDU	Controls
Male, n (%)	484 (82.7%)	663 (79.5%)
Age (years)	32.0 (26.0–39.0)	36.0 (31.0–42.0)
BD subgroups		
Complete BD, n	334	/
Incomplete BD, n	219	/
Severe BD, n	132	/
Un-severe BD, n	421	/
Early-onset BD, n	423	/
Late-onset BD, n	130	/
Phenotypes		
Uveitis	553	/
Genital ulcer	285	/
Erythema nodosum	233	/
Arthritis	250	/
Aphthous ulcer	545	/

BD, Behcet’s disease; BDU, Behcet’s uveitis.

Five hundred and fifty-three BD patients with detailed clinical data were included in the subgroup and phenotype association analysis.

### *ST2* rs3821204 SNP Was Associated With BD Development

*ST2* SNPs rs3821204, rs12712142, and rs2058660 were analyzed in both cases and controls and rs3821204 G allele was found to be associated with BDU development (X2 = 7.94, Pc = 0.039; **Table 2**). Although the frequency of GG genotype of *ST2* rs3821204 was higher in the control group than that in the BDU group (10.71 *vs* 6.16%), genotype frequency analysis failed to reach the statistical significance threshold as the trend was not statistically significant after Bonferroni correction (Pc = 0.051; **Table 2**). Logistic analysis with three models (i.e., additive, dominant, and recessive) revealed significant differences in the genotypic distributions of rs3821204 in both additive (Pc = 0.036) and recessive (Pc = 0.027) models (**Table 3**).

**Table 2:** Allele and genotype distribution of the IL-33 and ST2 gene markers in BDU patients and controls.

SNPs	Groups	Allele (%)		OR (95% CI)	χ^2^	P	Pc	Genotype (%)			χ^2^	P	Pc
		C	A					CC	CA	AA			
rs1891385	BDU	318 (27.3)	846 (72.7)	0.991 (0.838,1.172)	0.012	0.914	1	41 (7.0)	236 (40.5)	305 (52.4)	0.052	0.974	1
	Controls	456 (27.5)	1202 (72.5)					61 (7.4)	334 (40.3)	434 (52.4)			
		G	A					GG	GA	AA			
rs2210463	BDU	474 (40.7)	690 (59.3)	1.032 (0.886,1.202)	0.164	0.686	1	98 (16.8)	278 (47.8)	206 (35.4)	1.022	0.600	1
	Controls	665 (40.0)	999 (60.0)					145 (17.4)	375 (45.1)	312 (37.5)			
		C	T					CC	CT	TT			
rs11792633	BDU	537 (45.9)	633 (54.1)	1.118 (0.962,1.299)	2.103	0.147	1	125 (21.4)	287 (49.1)	173 (29.6)	2.397	0.302	1
	Controls	718 (43.1)	946 (56.9)					164 (19.7)	390 (46.9)	278 (33.4)			
		T	C					TT	TC	CC			
rs7044343	BDU	574 (49.1)	596 (50.9)	1.121 (0.965,1.302)	2.226	0.136	1	140 (23.9)	294 (50.3)	151 (25.8)	3.980	0.137	1
	Controls	770 (46.2)	896 (53.8)					192 (23.0)	386 (46.3)	255 (30.6)			
		G	A					GG	GA	AA			
rs1048274	BDU	588 (50.3)	580 (49.7)	1.136 (0.978,1.320)	2.779	0.096	0.764	148 (25.3)	292 (50.0)	144 (24.7)	4.247	0.120	0.957
	Controls	780 (47.2)	874 (52.8)					198 (23.9)	384 (46.4)	245 (29.6)			
		A	G					AA	AG	GG			
rs2058660	BDU	511 (43.7)	659 (56.3)	0.844 (0.726,0.980)	4.93	0.026	0.211	120 (20.5)	271 (46.3)	194 (33.2)	4.72	0.095	0.756
	Controls	797 (47.9)	867 (52.1)					201 (24.2)	395 (47.5)	236 (28.4)			
		G	C					GG	GC	CC			
rs3821204	BDU	321 (37.9)	526 (62.1)	0.790 (0.670,0.931)	7.94	0.005	0.039	36 (6.2)	249 (42.6)	299 (51.2)	10.12	0.006	0.051
	Controls	539 (48.0)	584 (52.0)					89 (10.7)	361 (43.4)	381 (45.8)			
		A	C					AA	AC	CC			
rs12712142	BDU	358 (44.2)	452 (55.8)	0.826 (0.704,0.970)	5.48	0.019	0.154	54 (9.2)	250 (42.8)	280 (47.9)	6.53	0.038	0.306
	Controls	580 (53.5)	504 (46.5)					112 (13.4)	356 (42.8)	364 (43.8)			

BDU, Behcet’s uveitis; OR, odds ratio; CI, confidence interval; χ2, Chi-square test; Pc, P values adjusted by Bonferroni correction, Pc = P × 8.

**Table 3:** Analysis of the eight SNPs based on three genetic models.

Gene	SNPs	Additive model				Dominant model				Recessive mode		
		**P**	**Pc**	**OR (95% CI)**		**P**	**Pc**	**OR (95% CI)**		**P**	**Pc**	**OR (95% CI)**
ST2	rs3821204	0.005	0.036	0.786 (0.665–0.928)		0.047	0.380	0.807 (0.653–0.998)		0.003	0.027	0.548 (0.366–0.819)
ST2	rs12712142	0.022	0.172	0.831 (0.710–0.973)		0.119	0.950	0.844 (0.683–1.044)		0.016	0.126	0.655 (0.465–0.924)
ST2	rs2058660	0.031	0.247	0.851 (0.735–0.985)		0.053	0.427	0.798 (0.634–1.003)		0.107	0.854	0.810 (0.627–1.046)
IL33	rs1891385	0.914	1	0.991 (0.836–1.173)		0.984	1	0.998 (0.807–1.234)		0.823	1	0.954 (0.633–1.439)
IL33	rs2210463	0.692	1	1.031 (0.888–1.197)		0.419	1	1.095 (0.879–1.365)		0.773	1	0.959 (0.724–1.271)
IL33	rs11792633	0.154	0.534	1.114 (0.961–1.292)		0.127	1	1.195 (0.951–1.502)		0.446	1	1.107 (0.852–1.437)
IL33	rs7044343	0.143	0.524	1.116 (0.963–1.293)		0.049	0.394	1.268 (1.001–1.607)		0.699	1	1.050 (0.819–1.347)
IL33	rs1048274	0.103	0.477	1.130 (0.976–1.310)		0.399	0.320	1.286 (1.012–1.635)		0.547	1	1.078 (0.844–1.378)

OR, odds ratio; CI, confidence interval; Pc, P values adjusted by Bonferroni correction, Pc = P × 8.

### *IL33* SNPs Were Not Associated With BD Development

We then detected *IL33* SNPs (rs11792633, rs1048274, rs7044343, rs2210463, and rs1891385) in the study population. Our study showed that neither genotype nor allele frequencies of these five SNPs were statistically significant between patients and healthy controls (Pc > 0.05; **Table 2**). Moreover, logistic regression analysis with three genetic models (i.e., additive, dominant, and recessive) also did not reveal any statistical difference between patient and control groups (Pc > 0.05; **Table 3**).

### Correlation of *ST2* and *IL33* SNPs With the Phenotype Subgroups of BD

Association analyses of *ST2* and *IL33* SNPs with different BD subgroups and phenotypes were also performed (**Tables 4A** and **4B**). The allele frequencies of *IL33* SNPs rs2210463 (P = 0.0028, Pc = 0.022), rs7044343 (P = 0.0034, Pc = 0.027), and rs1048274 (P = 0.0050, Pc = 0.040) were all significantly different between the complete-type and incomplete-type BDU groups. The allele frequency of *IL33* SNP rs2210463 (P = 0.006, Pc = 0.048) was significantly different between patients with and without genital ulcers. No allele frequency differences were found between the early- and late-onset or severe and non-severe BDU groups in any *ST2* or *IL33* SNP. Moreover, no association was found between erythema nodosum, or arthritis and *IL33* or *ST2* SNPs.

**Table 4A:** Analysis of eight SNPs based on different BD subgroups.

Gene	SNPs	Severe BD				Complete BD				Early-onset BD		
		**OR**	**95% CI**	**Pc**		**OR**	**95% CI**	**Pc**		**OR**	**95% CI**	**Pc**
ST2	rs3821204	0.897	0.657–1.225	1		0.799	0.609–1.049	0.845		0.735	0.545–0.993	0.354
ST2	rs12712142	1.070	0.796–1.439	1		0.951	0.734–1.233	1		0.708	0.529–0.948	0.161
ST2	rs2058660	1.141	0.865–1.506	1		1.066	0.836–1.358	1		0.844	0.639–1.116	1
IL33	rs1891385	0.995	0.728–1.359	1		1.132	0.864–1.483	1		1.098	0.799–1.509	1
IL33	rs2210463	0.693	0.518–0.926	0.102		0.684	0.533–0.878	0.022		0.823	0.621–1.091	1
IL33	rs11792633	0.839	0.635–1.109	1		0.760	0.596–0.969	0.213		0.849	0.643–1.121	1
IL33	rs7044343	0.801	0.607–1.058	0.941		0.697	0.547–0.887	0.027		0.925	0.700–1.221	1
IL33	rs1048274	0.138	0.614–1.070	1		0.707	0.555–0.901	0.040		0.895	0.677–1.182	1

BD, Behcet’s disease; OR, odds ratio; CI, confidence interval; Pc, P values adjusted by Bonferroni correction, Pc = P × 8.

**Table 4B:** Analysis of eight SNPs based on different phenotypes of BD patients.

	SNPs	Genital ulcer				Arthritis				Erythema nodosum		
		**OR**	**95% CI**	**Pc**		**OR**	**95% CI**	**Pc**		**OR**	**95% CI**	**Pc**
ST2	rs3821204	0.898	0.691–1.167	1		1.097	0.844–1.427	1		0.934	0.716–1.219	1
ST2	rs12712142	1.022	0.793–1.317	1		1.247	0.967–1.609	0.705		0.965	0.746–1.247	1
ST2	rs2058660	1.115	0.880–1.414	1		1.240	0.977–1.574	0.611		1.073	0.844–1.364	1
IL33	rs1891385	0.993	0.761–1.295	1		0.959	0.734–1.252	1		0.971	0.741–1.271	1
IL33	rs2210463	0.713	0.560–0.908	0.048		0.757	0.594–0.966	0.199		0.867	0.679–1.107	1
IL33	rs11792633	0.805	0.636–1.021	0.586		0.784	0.618–0.994	0.358		0.958	0.754–1.217	1
IL33	rs7044343	0.761	0.600–0.963	0.185		0.731	0.577–0.927	0.078		0.948	0.746–1.203	1
IL33	rs1048274	0.759	0.599–0.961	0.175		0.781	0.616–0.990	0.327		0.973	0.766–1.236	1

BD, Behcet’s disease; OR, odds ratio; CI, confidence interval; Pc, P values adjusted by Bonferroni correction, Pc = P × 8.

## Discussion

Although the etiopathogenesis of BD remains uncertain, microbial agent triggers, environmental factors, genetic predisposition, and immunological abnormalities have been implicated (8–10). Complex disorders implicating innate and adaptive immunity in humoral and cellular immunity settings are observed in BD. IL33, a member of the IL1 family that potently drives the production of T helper (Th)-2-associated cytokines and promotes responses by cytotoxic NK cells, Th1 cells, and CD8+ T cells (11), has been associated with various immune-related diseases (12, 13). ST2, the receptor of IL33, is highly expressed in many immunocytes (14).

In the present study, we demonstrated significant association between the *IL33/ST2* pathway SNP rs3821204 and the development of BDU. The rs3821204 polymorphism occurs in the 3′ untranslated region of *ST2*, which was found to be functional with the rs3821204 gene mutation by disrupting the binding site of miR-202-3p, resulting in plasma level changes of sST2 (15, 16). sST2 is the soluble form of ST2, which together with the membrane-bound form (ST2L), comprise the two main ST2 existing forms (17). ST2L functions as a transmembrane signaling receptor of IL33, mediating the effect of IL33 on inflammatory processes, while sST2 acts as a decoy receptor that prevents the interaction of ST2L with IL33 (18, 19).

Several studies have reported associations between *IL33* rs7044343 polymorphism and autoimmune diseases, including rheumatoid arthritis, systemic sclerosis, and BD in Turkey (20–22). Previous case-control study revealed a significant association between the rs7044343 TT genotype and Turkish BD patients in the discovery population (22). However, in the present study, rs7044343 SNP was not found to be associated with the development of BD in patients belonging to Chinese Han ethnicity. Ethnic differences may be a reason for this discrepancy, as genetic heterogeneity between races has been reported to be 58% in a previous research (23).

The association of *IL33/ST2* SNPs with different BD phenotype subgroups were also investigated. No associations were found except for genital ulcer and the BD of the complete-type. As compared with the other phenotype-based subgroups, greater genetic differences were found between the complete-type and incomplete-type BD groups, which indicated that different genetic backgrounds may exist in these groups of BD patients. Although previous studies reported that similar gene mechanisms underlie the pathogenesis of different immune diseases (24, 25), in the present study, rs2058660 [inflammatory bowel disease-associated (26)], rs1891385 [AS-associated (27)], and rs11792633 [systemic sclerosis-associated (28)] SNPs were not associated with either onset or phenotypes of BD. Compared with the classical immune diseases, BD presents more extensive pathological changes in tissues and organs with featured spontaneous exacerbation and remission clinical courses (29), which, together with the genetic findings, indicate that different molecular mechanisms may exist in BD pathology as compared to classical immune diseases.

Our study had several limitations. First, genetic variants may have different effects in diverse ethnicities. In this study, only Chinese Han individuals were recruited, thus, our results may not be generalizable to other ethnicities. Second, all the study subjects were enrolled from two tertiary ophthalmologic centers, therefore, selection bias may exist. Third, the mRNA expressions of BD patients with different SNP genotypes were not analyzed in this study, further studies are needed to validate our findings.

In conclusion, we reported the association of rs3821204, rs2210463, rs7044343, rs1048274 with the development or phenotypes of BDU, and for the first time revealed significant genetic differences between patients with complete-type and incomplete-type BD. Our findings indicate that *IL33/ST2* polymorphisms have a close and probable causal association with BDU, and different genetic backgrounds may exist in complete-type and incomplete-type BD patients.

## Data Availability Statement

The raw data supporting the conclusions of this article will be made available by the authors, without undue reservation.

## Ethics Statement

The studies involving human participants were reviewed and approved by The Ethics committee of Peking Union Medical College Hospital. Written informed consent to participate in this study was provided by the participants/participants’ legal guardian/next of kin.

## Author Contributions

MP participated in the study design, patient enrollment, data collection and analysis and manuscript drafting. MZ participated in the study design, patient enrollment, data collection and analysis, manuscript drafting, and coordination of experimental work. XL participated in the data interpretation and analysis. PY, CZ, and FG participated in the patient enrollment. MP, YQ, AL, and JX carried out the experimental work. All authors contributed to the article and approved the submitted version.

## Funding

This work was supported by the National Natural Science Foundation of China [81770917] (fees for patient enrollment, carrying out the experimental work and open access publication) and Beijing Municipal Science and Technology Commission No. Z171100001017217 (fees for patient enrollment).

## Conflict of Interest

The authors declare that the research was conducted in the absence of any commercial or financial relationships that could be construed as a potential conflict of interest.
